# Assessment of Vertebral Body Bone Quality in Adolescent Idiopathic Scoliosis (AIS) Using Micro‐CT, qBEI, and Nanoindentation—A Case Study

**DOI:** 10.1002/jsp2.70217

**Published:** 2026-09-01

**Authors:** Natalia M. Castoldi, Edmund Pickering, Jiaqiu Wang, Madge Martin, James Roberts, Jurgis Ruza, Laure Stickel, Geoffrey N. Askin, Robert D. Labrom, Maree T. Izatt, Davide Fontanarosa, J. Paige Little, Peter Pivonka

**Affiliations:** ^1^ School of Mechanical, Medical and Process Engineering Queensland University of Technology Brisbane Australia; ^2^ Centre for Biomedical Technologies Queensland University of Technology Brisbane Australia; ^3^ Faculty of Mechanical Engineering Technische Hochschule Nürnberg Georg Simon Ohm Nuremberg Germany; ^4^ School of Computer Science and Digital Technologies London South Bank University London UK; ^5^ MSME UMR 8208, Univ Paris Est Creteil, Univ Gustave Eiffel, CNRS Creteil France; ^6^ Biomechanics and Spine Research Group Queensland University of Technology Brisbane Australia; ^7^ Department of Material Science and Engineering Massachusetts Institute of Technology Cambridge USA; ^8^ Queensland Children's Hospital Brisbane Australia; ^9^ School of Clinical Sciences Queensland University of Technology Brisbane Australia

**Keywords:** adolescent idiopathic scoliosis, bone quality, microCT, nanoindentation, quantitative backscattered electron imaging, vertebral body

## Abstract

**Background:**

Adolescent idiopathic scoliosis (AIS) is the most common three‐dimensional spinal deformity, predominantly affecting females aged 10–16 years. Although no single etiological factor has been definitively identified, alterations in bone quality and biomechanical properties have been proposed as contributing factors to AIS development.

**Methods:**

We developed a surgical protocol to obtain trabecular bone biopsies from the apex vertebra during anterior corrective surgery for AIS. Multimodal analysis was performed on samples from eight AIS participants (14.8 ± 1.5 years), including microstructural assessment via micro‐computed tomography (micro‐CT), bone mineral density distribution (BMDD) via quantitative backscattered electron imaging (qBEI), and mechanical characterization via nanoindentation. Correlations with major curve angle, apical wedge angle, and Risser grade were investigated.

**Results:**

Mean trabecular bone volume fraction (BV/TV) was 0.146 ± 0.029, comparable to values reported in healthy populations. Mean trabecular thickness (Tb.Th) was 0.146 ± 0.011 mm, suggesting slightly thicker trabeculae than typically reported in healthy young cohorts. BMDD analysis revealed CaPeak values of 23.34 ± 1.02 wt% Ca, and greater CaWidth values (4.26 ± 0.33 wt% Ca) compared to healthy adolescent and adult reference data. CaPeak was significantly negatively correlated with apical wedge angle, indicating lower mineralization with increasing vertebral wedging. Nanoindentation showed a negative association between reduced modulus (13.37 ± 2.5 GPa) and apical wedge angle, with hardness values of 0.412 ± 0.079 GPa.

**Conclusions:**

These findings provide novel insights into vertebral trabecular bone alterations in AIS and their association with deformity severity. To our knowledge, this is the first study to integrate micro‐CT, qBEI, and nanoindentation analyses in AIS vertebral biopsies, revealing structural, compositional, and mechanical differences that may contribute to the pathogenesis and progression of AIS.

## Graphical abstract

A new biopsy protocol enabled the first combined micro‐CT, qBEI, and nanoindentation study of AIS vertebral bodies, revealing slightly thicker trabeculae, broader mineralization distribution, and decreasing mineralization and reduced modulus with vertebral wedge angle severity—supporting a role for altered bone quality in AIS.

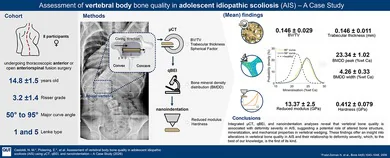

## 1 Introduction

Adolescent idiopathic scoliosis (AIS) represents the most prevalent three‐dimensional spinal deformity, predominantly affecting females aged 10–16 years [1]. The condition occurs in 0.5%–5.2% of the general population [1, 2]. Without treatment, severe spinal curves can double mortality risk through complications including chronic back pain, cardiopulmonary dysfunction, and mental health deterioration [3]. Although the precise etiology of AIS remains unclear, several hypotheses have been proposed, including bipedal posture affecting spinal stability [4], genetic predisposition [5, 6], abnormal growth patterns with earlier peak growth velocity and more slender spines [4, 7, 8], and intervertebral disc swelling from reduced spinal loading [4, 9, 10, 11].

Biomechanically, asymmetrical paraspinal muscle volume at the apex vertebra [12, 13] and altered bone quality—including systemic osteopenia [14, 15] and impaired bone microarchitecture [16, 17]—have been proposed as contributing factors. However, no single factor has been conclusively identified as the cause of scoliosis, and determining its etiology remains a challenge. This is partly because children and adolescents are still growing, with many simultaneous changes occurring in their bodies, making it difficult to isolate specific factors. Obtaining experimental data from AIS patients is also difficult, especially when it comes to bone quality.

Despite these challenges, altered bone quality in AIS has motivated extensive investigation of bone micro‐architectural properties. Studies using in vivo imaging (HR‐pQCT, DEXA) have revealed that AIS predominantly affects trabecular volumetric bone mineral density (vBMD) and microstructure [18], with comprehensive abnormalities including altered endocortical modeling, disrupted trabecular structure, and disturbed mineralization [19], altered trabecular number and separation patterns [20], with associations between curve progression and increased bone turnover [21] and low BMD as a potential prognostic factor [22]. Bone biopsy studies provided more detailed micro‐architectural analysis, revealing significantly lower lumbar and femoral neck BMD [23]; and reduced trabecular number and connectivity, predominant changes in trabecular rods, and decreased mineralization in the iliac crest [16]. Spinous process biopsies during posterior fusion surgery show decreased bone resorption and formation rates [17, 24], suggesting that low bone turnover may contribute to AIS pathogenesis, consistent with findings that reduced bone mass in the hip and spine represents a significant risk factor for curve progression [25].

However, no study has examined the vertebral body itself, despite its exposure to highly asymmetrical mechanical loading, particularly at the apex vertebra, where loads are substantially higher on the concave than on the convex side. Such asymmetry may induce spatial variations in vertebral bone density [26], as demonstrated in animal models [27, 28]. This gap largely reflects the limited access to vertebral bodies, which requires infrequently performed anterior surgical approaches [29, 30, 31, 32, 33, 34]. Although Adam and Askin [35] used low‐dose CT to identify convex–concave BMD differences at the apex, the resolution was insufficient to assess trabecular microarchitecture. Moreover, no studies have investigated bone mineral density distribution (BMDD), a tightly regulated property independent of age, sex, and ethnicity in healthy individuals [36, 37]. This gap persists because HR‐pQCT cannot reliably quantify BMDD due to beam‐hardening artifacts, and bone biopsies—required for BMDD assessment—are rarely obtained in pediatric AIS patients.

To address these gaps, we designed a surgical protocol for acquiring trabecular bone samples from the vertebral body during anterior approach correction, offering a unique opportunity to collect biopsies directly from the vertebral body prior to screw insertion. To our knowledge, this is the first study analyzing vertebral body biopsies from AIS patients at correction surgery. We evaluated bone quality using three complementary methods: micro‐CT to characterize trabecular microarchitecture, including bone volume fraction (BV/TV), trabecular thickness (Tb.Th), and ellipsoid factor (Tb.EF); quantitative backscattered electron imaging (qBEI) to measure BMDD; and nanoindentation to assess microscale mechanical properties, including reduces modulus and hardness. Finally, we examined relationships between these parameters and major curve angle, Risser grade, and apical wedge angle: major curve angle to assess relationships with scoliosis severity, Risser grade to determine whether variations in these parameters are due to age differences, and apical wedge angle to assess whether there is any relationship to the specific deformation of the vertebral bone, since major curve angle, especially in the lumbar spine, may be mainly due to disc wedging rather than vertebral wedging [38].

## 2 Materials and Methods

### 2.1 Patient Population

Eight female AIS patients were recruited between 2018 and 2020 from the Queensland Children's Hospital Spine Deformity Clinic, Brisbane, Australia (with parental/guardian consent). Inclusion criteria: (1) AIS diagnosis, (2) right thoracic (Lenke type 1) or left thoracolumbar (Lenke type 5) major curve [39], (3) clinical indication for anterior approach surgery (thoracoscopic or open anterior spinal fusion) for progressive scoliosis. Preoperative clinical data included coronal/sagittal curve angles, Risser grade from standing radiographs [40], and angle of trunk rotation measured with a Scoliometer. Study participation did not affect clinical care. Patients provided vertebral bone cores from the curve apex and had the following demographics: age 14.8 ± 1.5 years; height 164.4 ± 7 cm; weight 50.9 ± 7.5 kg; BMI 18.8 ± 1.9 kg/m^2^; Risser grade 3.2 ± 1.4. Participant details can be found in Table 1.

**TABLE 1:** Summary of participant information, including age, Risser grade, Lenke classification, apex vertebra, and major curve angle.

Participant	Age (years)	Risser grade	Lenke type	Apex vertebra	Major curve angle (°)
**●**	16.18	2	Type 5	T12	80
2 **◄**	16.91	5	Type 1	T10	50
3 **■**	12.84	1	Type 5	T12	95
4 **►**	14.63	4	Type 5	L1	55
5 **♦**	15.78	4	Type 5	T12	65
6 **★**	12.52	1	Type 1	T9	69
7 **▼**	14.75	4	Type 1	T8	64
8 **▲**	15.05	3	Type 1	T8	60

*Note:* Colored symbols correspond to markers used in the figures.

Participant and parent/guardian informed‐consent was obtained under both Hospital and University ethical approvals from Children's Health Queensland Human Research Ethics Committee (HREC) (2018/QCHQ/45601) and the Queensland University of Technology HREC (1800001128).

### 2.2 Apical Wedge Angle Assessment

From the pre‐surgical radiographs, the apical coronal wedge angle was measured according to the method of Cobb [41], defined as the angle between the superior and inferior endplates of the apical vertebra. Measurements were performed using ImageJ (version 1.54) [42]. Two raters each performed three independent measurement sessions. All measurements were repeated on separate days (minimum interval of 5 days), with the radiographs presented in a different order for each session. Raters were blinded to their previous measurements to minimize recall bias. Intra‐ and inter‐rater reliability were assessed using the intraclass correlation coefficient (ICC(A,1)) [43] and the mean absolute difference. Intra‐rater reliability was excellent, with ICC(A,1) values of 0.94 and 0.96 for Raters 1 and 2, respectively. Inter‐rater reliability was also excellent (ICC(A,1) = 0.992), with a mean absolute difference of 0.47°.

### 2.3 Surgical Procedure and Bone Core Retrieval

**FIGURE 1:**
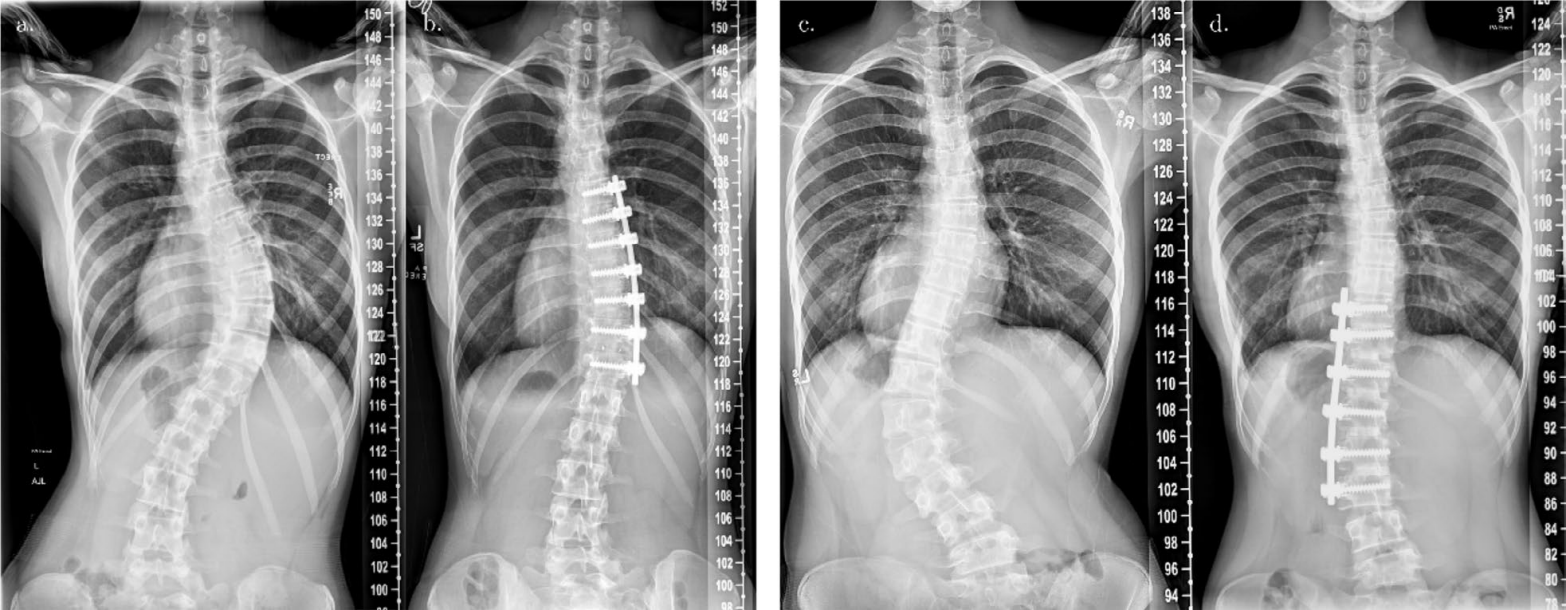
Representative X‐ray images showing scoliosis correction outcomes. (a) Preoperative major thoracic curve angle of 55°, (b) post‐operative curve angle of 21° following thoracoscopic anterior scoliosis correction surgery (T6–T12). (c) Pre‐operative major thoracolumbar curve angle of 57°, (d) post‐operative curve angle of 20° following open anterior scoliosis correction surgery (T10–L3).

**FIGURE 2:**
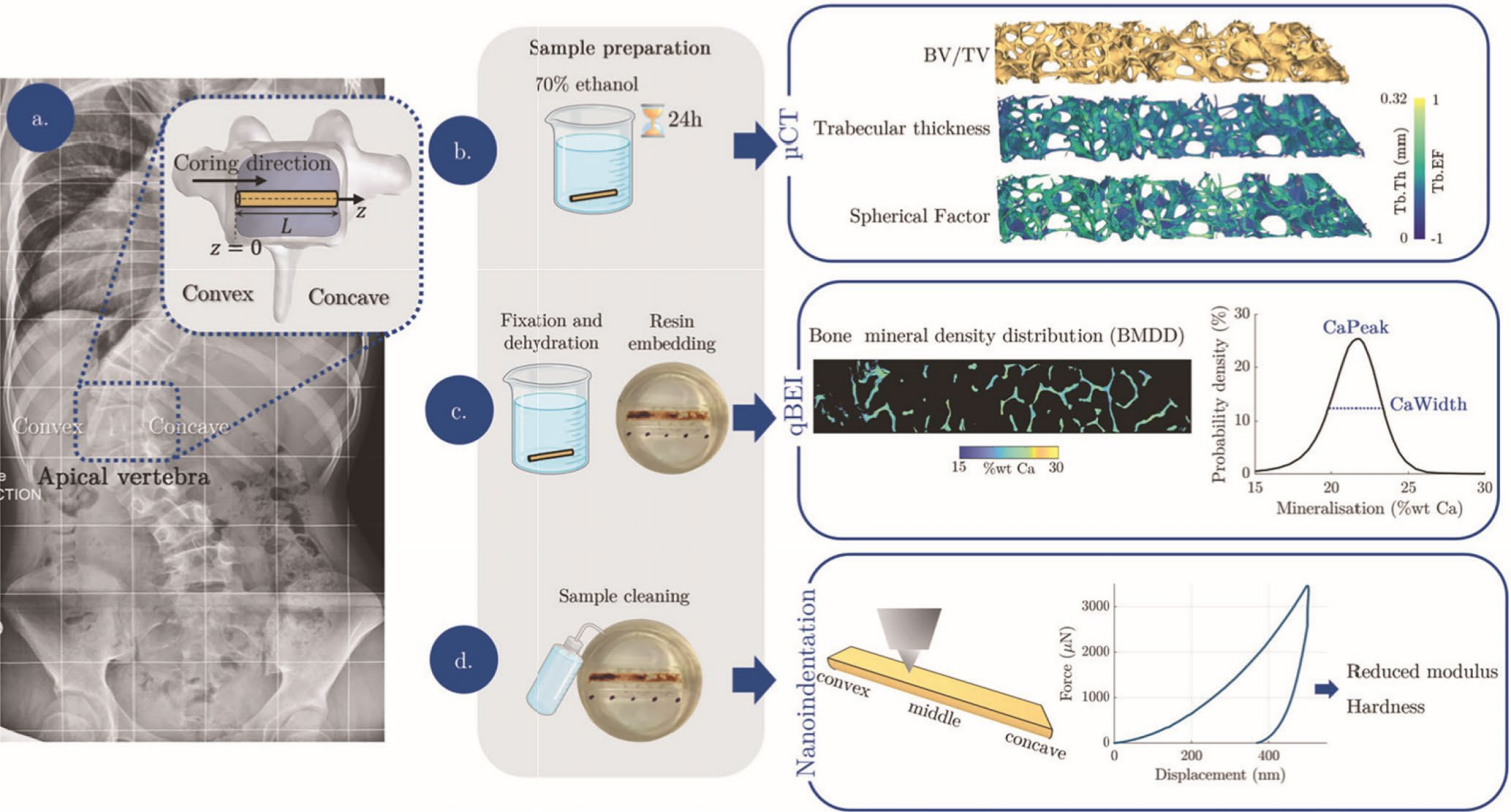
Schema of experimental methodology: (a) X‐ray of a single AIS thoracolumbar curve with convex/concave sides identified together with schematic rendering of the apex vertebral body from which bone cores are surgically extracted. A biopsy needle was inserted through the trabecular bone of the convex side to the concave side. The concave side of the specimen is then cut at approximately 45° to simplify identification of the specimen. (b) Bone specimens were placed into a 70% ethanol solution for 24 h. Subsequently, they underwent micro computed tomography (micro‐CT) imaging, where further analysis of bone volume fraction (BV/TV), trabecular thickness (Tb.Th), and ellipsoid factor (Tb.EF) were performed. (c) Samples were then fixed in an ethanol solution (70%) and dehydrated in a graded series of ethanol (maximum concentration 100% for 24 h) before being resin infiltrated in polymethylmethacrylate, and coated with 20 nm carbon. Samples were observed under scanning electron microscopy, and backscatter panoramas were acquired to analyze bone mineral density distributions (BMDD) and related parameters, such as CaPeak and CaWidth. (d) Samples were cleaned of carbon using ethanol and mounted into the nanoindenter, where 15 indentations were performed, divided into three regions: Convex, middle, and concave; resulting in a force‐displacement curve to analyze reduced modulus and hardness.

All anterior spinal fusion surgeries and bone core retrievals were performed by experienced spine orthopedic surgeons during scheduled scoliosis instrumented fusion procedures. The anterior approach provides direct access to thoracic or lumbar vertebral bodies prior to screw insertion (see Figure 1). Two techniques were used depending on curve location: a thoracoscopic anterior approach for major thoracic curves, requiring single‐lung deflation, and a lateral subcostal incision for major thoracolumbar curves. These approaches uniquely enabled vertebral body biopsy immediately before screw placement, unlike the more common posterior approach. Vertebral levels at or near the apex of the major scoliotic curve were identified on standing radiographs in AIS patients. Throughout, “concave” and “convex” denote the respective sides of the scoliosis curve in the coronal plane (see Figure 2).

Cylindrical bone cores were extracted from the convex side of the selected apical vertebra at midvertebral height using a T‐Lok 8G (6‐in.) biopsy needle (Argon Medical Devices, Texas), at the planned vertebral screw location during instrumented surgical correction. Under image‐intensifier guidance, the needle was advanced towards the concave side of the vertebral body. This biopsy system preserves sample integrity by capturing and retaining bone without alteration. A preliminary, unpublished study on ovine vertebrae confirmed that biopsy extraction did not affect screw pullout strength, informing the choice of needle size. The retrieved cores had a diameter of 3.8 mm and lengths between 10 and 20 mm. Cores were essentially bicortical, with length variation reflecting the integrity of these fine‐gauge samples during extraction.

To facilitate specimen orientation, a 45° bevel cut was made at the concave end (see Figure 2), enabling unambiguous identification of the longitudinal direction of the cylindrical bone samples. Accordingly, bone core position is defined with *z* = 0 at the convex side, and the z‐axis increasing from convex to concave, as shown in Figure 2.

### 2.4 Micro Computed Tomography (Micro‐CT) Scanning

After extraction, bone specimens were placed into a 70% ethanol solution for 24 h and subsequently underwent micro‐computed tomography (micro‐CT) imaging (Scanco Medical 50) at 6.8 μm resolution, 70 kVp, 114 μA, and 0.5 Aluminum filter. The micro‐CT images were preprocessed to eliminate artifacts of centroid drift of micro‐CT slices using a custom MATLAB (MathWorks) code. The plane coordinates of each 2D slice were corrected by aligning the barycenter of each slice in a line, and the sequence was reconstructed as a cuboid volume. Subsequently, the volume was binarized based on the voxel intensity using the Otsu method [44]. To estimate a representative volume of interest (VOI), a cylindrical VOI was defined with the center along the bone specimen's cylindrical axis and a given radius R. The voxels whose Euclidean distance to the centroid was less than the given radius R were included within the VOI. The number of slices used in the analysis varied with the specimen length, ranging from 1286 to 3357 slices. The analysis performed in this VOI consisted of: BV/TV; ellipsoid factor (Tb.EF); and Tb.Th (Figure 2).

**FIGURE A1:**
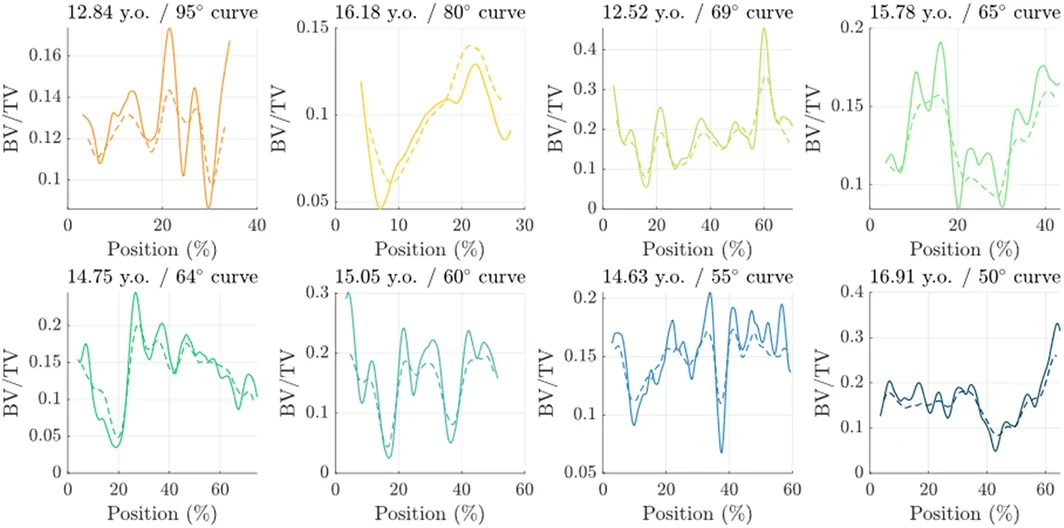
Bone mineral density evolution (BV/TV) of vertebral body bone specimens versus normalized specimens' length. Solid lines stand for the VOI radius size of *R* = 1 mm, and dashed lines for the VOI radius size of *R* = 1.5 mm. Note that the graphs are ordered by the value of curve angle, and the colors can be matched to the participants as described in Table 1.

The BV/TV was measured from the acquired micro‐CT imaging data using a custom MATLAB (MathWorks) code. BV/TV was calculated as the algebraic sum of intensity of the VOI bone voxels divided by the total number of voxels in the VOI. We first investigated changes in the VOI radius stepwise from the minimum R of 6.8 μm to the maximum R of 1.9 mm, that is, the approximate radius of the bone specimen. A radius R of 1.0 mm (150 voxels) was determined to give the best results, as this balanced the need for a larger VOI for representative measurements of bone matrix and marrow spaces, while not being so large as to be impacted from edge effects due to the biopsy needle extraction process (see results in Figure A1 in Appendix A), and this VOI radius was used on the following analysis.

Tb.Th and Tb.EF were extracted from micro‐CT imaging data using BoneJ [45]. Tb.EF was measured to study the rod/plate geometry of the trabeculae, by fitting ellipsoids at each point and measuring how plate‐like or rod‐like they are, using a scale from −1 to 1, respectively. The algorithm seeds ellipsoids along the trabecular medial axis and optimizes their size through iterative shape adjustments [46, 47]. Tb.Th was measured at each point as the diameter of the largest sphere fitting inside the structure containing that point [48].

### 2.5 Quantitative Backscattered Imaging (qBEI)

Following micro‐CT scanning, samples were fixed in an ethanol solution (70%) and dehydrated in a graded series of ethanol (maximum concentration of 100% for 24 h) before being resin infiltrated in polymethylmethacrylate (Technovit, Kulzer) [49]. Samples were ground to expose the midplane of the bone core and coated with 20 nm carbon. Samples were observed under scanning electron microscopy (Tescan Tima, resolution 1 μm, 20 kV, spot size 67 nm), and backscatter panoramas were acquired (pixel resolution of 1 μm), see Figure 2.

The BMDD was measured using quantitative backscattered imaging (qBEI) [49] with carbon and SiO_2_ standards for calibration. The following quantities of the calcium weight percent BMDD frequency plots were extracted from these curves: (i) calcium peak (CaPeak), that is, the value of Ca weight % at peak of the BMDD frequency curve; and (ii) calcium width (CaWidth), that is, width of the BMDD frequency curve at half of the peak BMDD [50] (see Figure 2 for detail).

Note that in a preliminary, unpublished study on ovine vertebrae, bone samples were scanned at our facility and subsequently re‐scanned at the Ludwig Boltzmann Institute of Osteology (Vienna, Austria), the same facility that acquired the qBEI data from young [37] and adult [49] human samples used as reference values in the present study. Ovine qBEI measurements obtained at both sites showed concordant results for the ovine specimens, supporting the comparability of the present AIS samples with healthy control data.

### 2.6 Nanoindentation

Following the qBEI analysis, samples were cleaned of carbon using ethanol and mounted into the nanoindenter (Hysitron TI 950 Triboindenter, diamond Berkovich indenter, tip diameter of 100 nm). Indentation points were selected on the bone at the concave, middle, and convex sites (*n* = 5 per site). Samples were loaded at a rate of 20 nm/s to a depth of 500 nm, held for 60 s, and then unloaded at a rate of 20 nm/s. Reduced modulus and hardness were determined via the Oliver‐Pharr method using the inbuilt software [51]. For reference, Figure 2 shows a typical force‐displacement curve from nanoindentation, demonstrating a typical Oliver‐Pharr [51] like curve.

### 2.7 Data Analysis

Given the small sample size (*n* = 8) and the ordinal nature of the Risser grade, Spearman's rank correlation coefficient (*ρ*) was used to assess associations between clinical and microstructural variables. Kendall's tau‐b was additionally computed as a robustness check for associations involving Risser grade. Experimental variables (BV/TV, Tb.Th, Tb.EF, reduced modulus, hardness, CaPeak, CaWidth) were evaluated against age, Risser grade, apical coronal wedge angle and major curve angle. Correlations among the experimental variables were also assessed. For our sample size (*N* = 8), correlations were considered significant at *p <* 0.05.

## 3 Results

Mean values along the bone length from the three analyses (micro‐CT, qBEI, and nanoindentation) are presented here. No clear or consistent trends in the measured properties were observed along the specimen length. Detailed raw data are provided in Appendix A. For simplicity, “major curve angle” will be referred to as “curve angle”, and the apical coronal wedge angle will be referred to as “apical wedge angle”.

### 3.1 Microstructure

**FIGURE 3:**
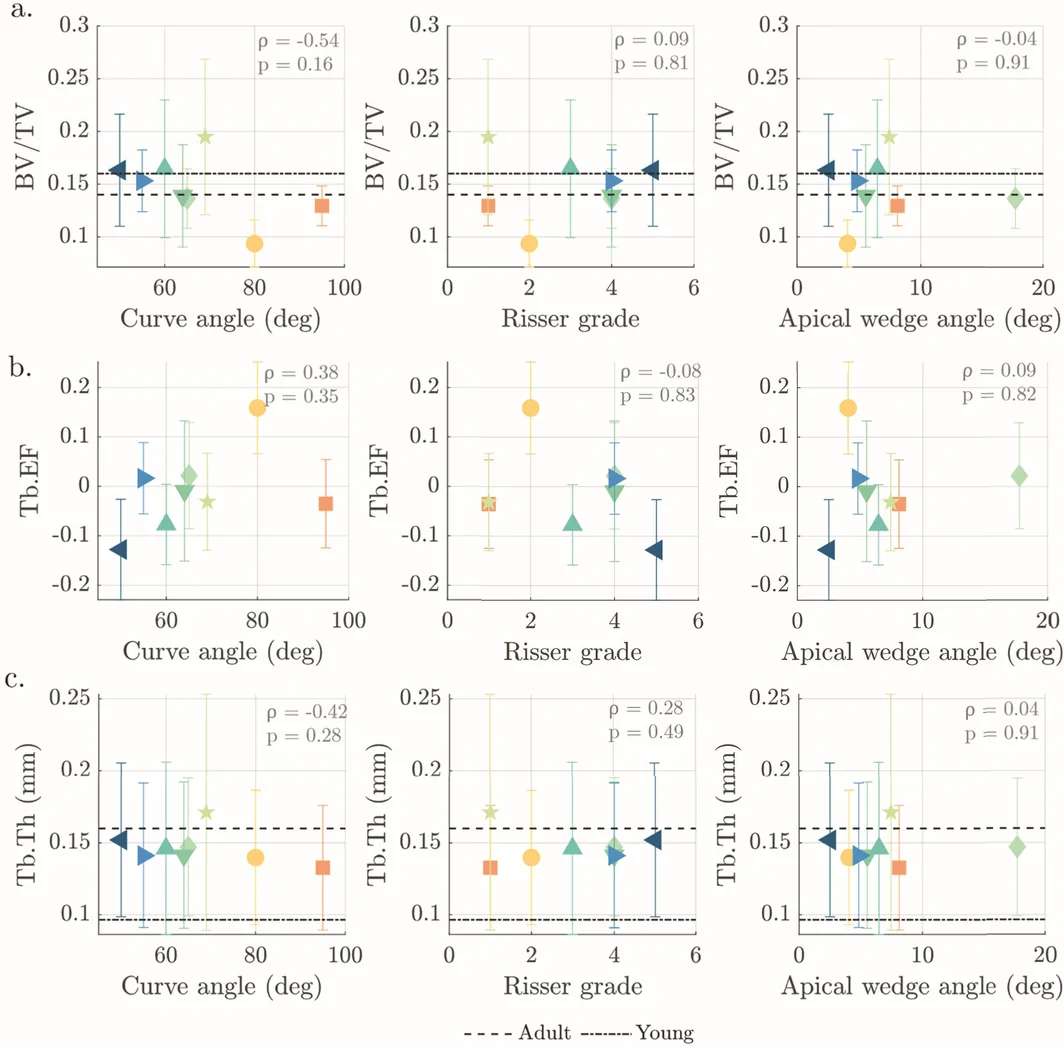
Relationship between curve angle (left), Risser grade (middle) and Apical wedge angle (right) versus average over the apical cores of (a) BV/TV (b) Tb.EF, and (c) Tb.Th, with reference values for Adult [52] (dashed line) and Young [16, 23] (dot‐dashed line) iliac crest bone cores. Spearman's *ρ* and the corresponding *p*‐value are shown in gray in each panel. Each marker and color consistently represent the same participant across plots. Error bar stands for the standard deviation.

We observe relatively constant BV/TV values, in good agreement with those reported for healthy iliac crest bone in adult [52] and young [16, 23] populations. No significant associations were observed with curve angle, Risser grade, or apical wedge angle (Spearman's *p* > 0.1), as shown in Figure 3a. The ellipsoid factor (Tb.EF) exhibited greater variability, but no significant correlations were observed with any of these parameters (Figure 3b). Mean Tb.Th also appeared relatively constant across patients, with values higher than those typically reported for the iliac crest in young individuals [16, 23] (Figure 3c) and more closely resembling those reported in adults [52]. Tb.Th likewise showed no clear dependence on curve angle, Risser grade, or apical wedge angle (Spearman's *p* > 0.2).

### 3.2 Mineral Distribution

**FIGURE 4:**
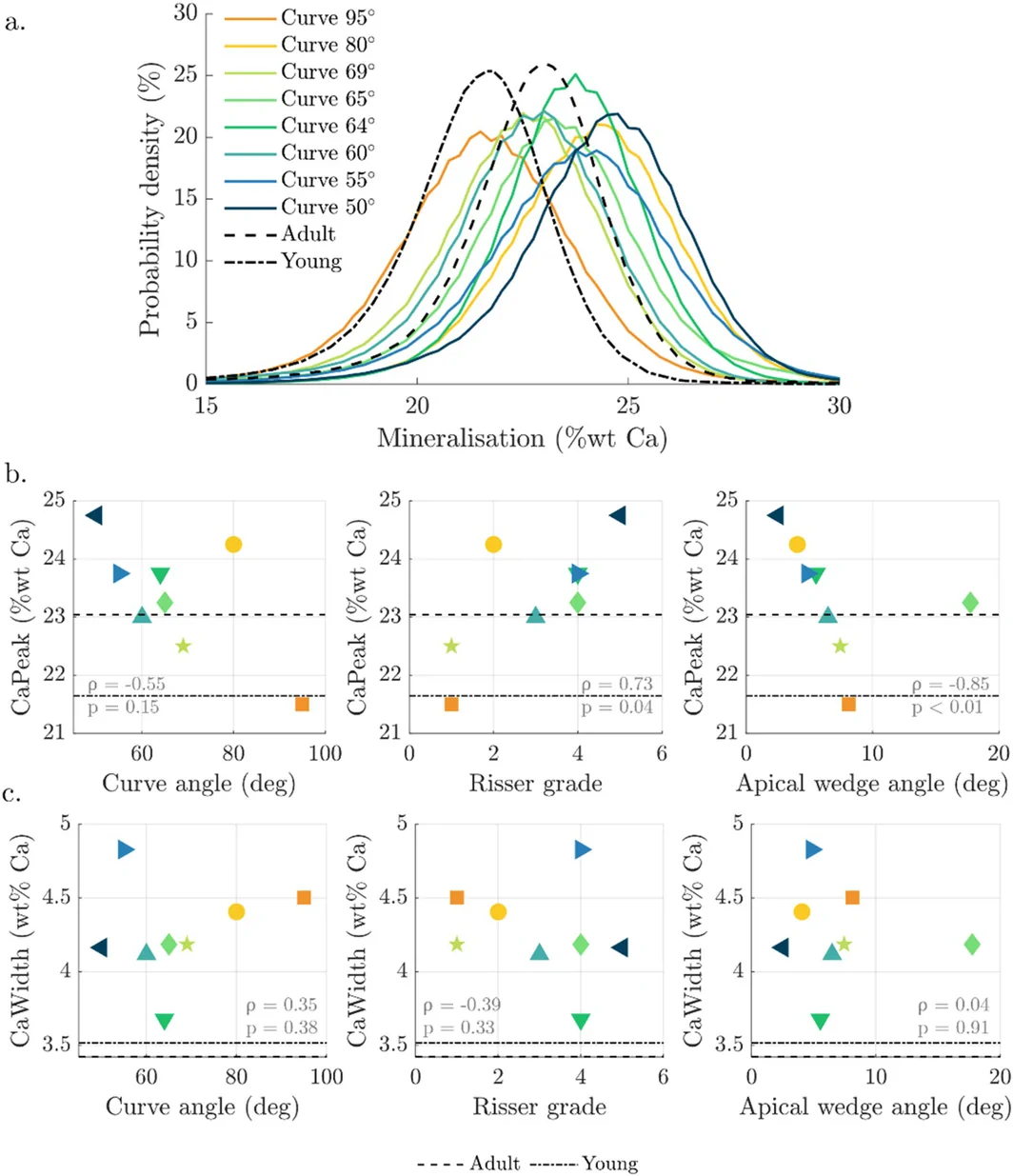
(a) Mean bone mineral density distribution (BMDD) plots for each AIS subject bone core (averaged over specimen length). Relationship between curve angle (left), Risser grade (middle), and Apical wedge angle (right) versus (b) mineralization peak (CaPeak) (c) BMDD width (CaWidth). All results are shown against reference BMMD from healthy children and adolescence [37] (labeled as young in dot‐dashed lines) and healthy adult transiliac trabecular bone cores [49] (dashed lines). Spearman's *ρ* and the corresponding *p*‐value are shown in gray in each panel. Each marker and color consistently represent the same participant across plots.

Figure 4a shows BMDD curves for all 8 AIS participants alongside those from iliac crest biopsies of healthy young [37] and adult [49] subjects. Most AIS participants exhibited higher peak calcium concentration (wt% Ca) than healthy children/adolescents (Figure 4b), often exceeding typical adult levels. CaPeak showed a non‐significant correlation with major curve angle (Spearman's *ρ* = −0.55, *p* = 0.15) but a strong negative correlation with apical wedge angle (Spearman's *ρ* = −0.85, *p* < 0.01), indicating that higher apical wedge angles were associated with lower mineralization. Moreover, CaPeak was positively correlated with Risser grade (Spearman's *ρ* = 0.73, *p* = 0.04; Kendall's *τ*_*b*_, *p* = 0.038), as shown in Figure 4b. AIS bone cores also showed greater mineral content heterogeneity, indicated by wider full width at half maximum (CaWidth) of the BMDD curve [49] (Figure 4c). CaWidth exceeded values in both healthy children/adolescents and adults, although no significant correlations with curve angle, Risser grade, and apical wedge angle were observed.

### 3.3 Mechanical Properties

**FIGURE 5:**
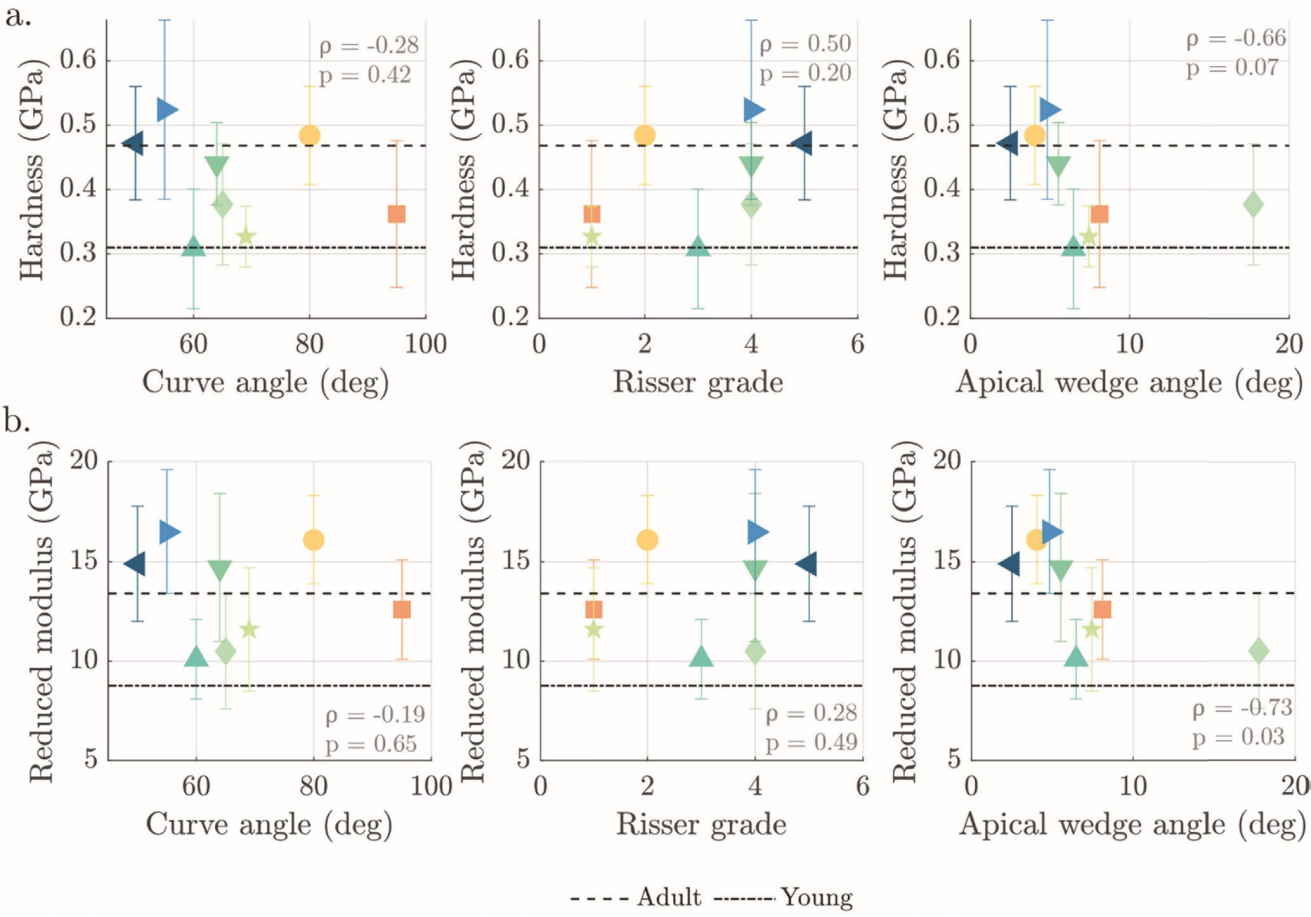
Relationship between curve angle (left), Risser grade (middle), and apical wedge angle (right) versus the average over the apical cores of (a) hardness, (b) reduced modulus. All results are shown against values from nanoindentation analysis of cranial bone in healthy children and adolescence [53] (labeled as young in dot‐dashed lines) and healthy vertebral bone in adults [54] (labeled as adult in dashed lines). Spearman's *ρ* and the corresponding *p*‐value are shown in gray in each panel. Each marker and color consistently represent the same participant across plots. Error bar stands for the standard deviation.

Mean hardness among participants was in good agreement with values reported for both young and adult populations, as shown in Figure 5a. A trend towards a negative correlation was observed between hardness and apical wedge angle (Spearman's *ρ* = −0.66, *p* = 0.07), while no significant correlations were observed with major curve angle (Spearman's *ρ* = −0.28, *p* = 0.42) or Risser grade (Spearman's *ρ* = 0.50, *p* = 0.22). Similarly, reduced modulus values were in good agreement with those reported for young and adult populations (Figure 5b). A significant negative correlation was observed between reduced modulus and apical wedge angle (Spearman's *ρ* = −0.73, *p* = 0.03), while no significant correlations were observed with major curve angle (Spearman's *ρ* = −0.19, *p* = 0.65) or Risser grade (Spearman's *ρ* = 0.28, *p* = 0.49).

### 3.4 Relationship Among Experimental Variables

**FIGURE 6:**
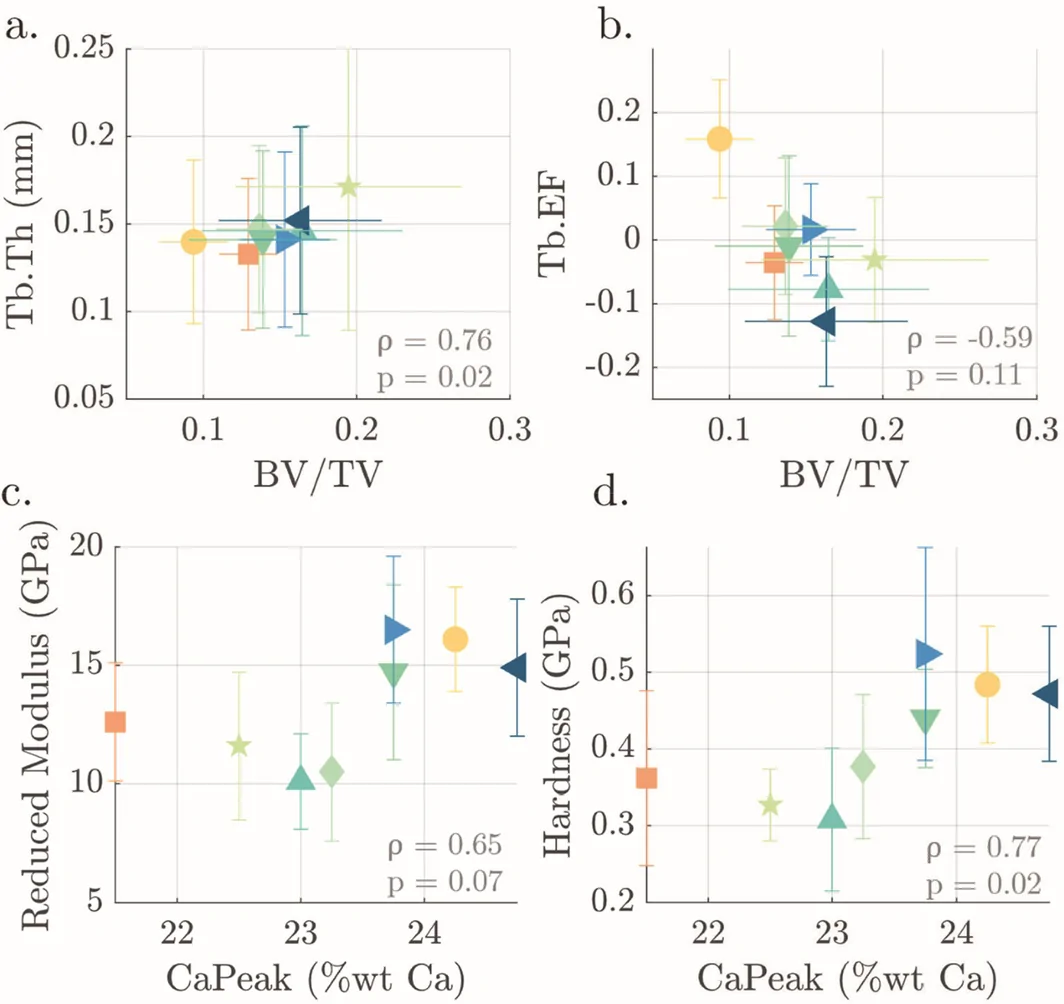
Associations among experimental variables. Relationships among the micro‐CT experimental variables are shown for BV/TV versus (a) Tb.Th and (b) Tb.EF. Associations between qBEI and nanoindentation measurements are shown for CaPeak versus (c) reduced modulus and (d) hardness. Spearman's *ρ* and the corresponding *p*‐value are shown in gray in each panel. Each marker and color consistently represent the same participant across panels. Error bars represent the standard deviation.

Some of the experimental variables showed associations with one another. In the micro‐CT analysis, Tb.Th was positively correlated with BV/TV (Spearman's *ρ* = 0.76, *p* = 0.02), as shown in Figure 6a, while Tb.EF showed a trend towards a negative correlation with BV/TV (Spearman's *ρ* = −0.59, *p* = 0.11), as shown in Figure 6b. Associations were also observed between the qBEI and nanoindentation measurements. Reduced modulus showed a trend towards a positive correlation with CaPeak (Spearman's *ρ* = 0.65, *p* = 0.07), as shown in Figure 6c, while hardness was positively correlated with CaPeak (Spearman's *ρ* = 0.77, *p* = 0.02), as shown in Figure 6d. These findings suggest that higher mineralization was associated with greater tissue‐level mechanical properties, as reflected by increased hardness and, to a lesser extent, reduced modulus.

## 4 Discussion

In this work, we investigated the bone quality and mechanical properties of bone cores from the vertebral bodies of AIS patients sampled during their scoliosis correction surgery performed via an anterior approach. This study presents a unique dataset, as the majority of scoliosis correction surgeries are performed posteriorly, which do not provide access to the vertebral body. To the best of the authors' knowledge, this is the first study to analyze the trabecular bone of the vertebral body in patients with AIS.

BV/TV in AIS bone biopsies ranged from 10% to 17.5%, with no correlation to curve angle or apical wedge angle. Although biopsy extraction may have biased trabecular spacing by breaking trabeculae, our measurements were consistent with other studies across populations, ages, and bone sites (Table 2). Specifically, micro‐CT analysis of AIS iliac crest biopsies showed 18%–19.9%, while in vivo measurements at the distal radius reported 12.4%–13%. Our results were also consistent with previously reported BV/TV values in a healthy young population obtained using micro‐CT of the iliac crest [16, 23] and HR‐pQCT of the distal radius (12.7%–12.8% ± 2.1%) [19, 20]. However, our reported BV/TV values were lower than those reported by Glorieux et al. [55], who analyzed iliac crest biopsies of healthy adolescents (14–16.9 years) using histomorphometry and reported a BV/TV of 25.7% ± 5.3%. Notably, our values are closer to those reported in older adults, including 13.5% (range: 7.8%–18.7%) in T9 vertebrae [56] and approximately 14% at the iliac crest [52]. These findings suggest no apparent correlation between AIS and BV/TV. When analyzed in relation to Risser grade, no variation in BV/TV was observed. Therefore, bone changes due to growth do not appear to be the predominant mechanism driving BV/TV variations in vertebral bodies of those with AIS. This finding aligns with the analysis by Mitchell et al. [57], who reported a constant total BV/TV evolution with increasing bone age. Regarding spatial variation of BV/TV across the apex vertebral body, we found no consistent trend (i.e., monotonously increasing or decreasing BV/TV from convex to concave side) for all analyzed specimens (Figure A1). Based on these results, we conclude that region‐dependent bone adaptation (convex versus concave) is not a significant mechanism in AIS. However, this finding should be interpreted with caution, given the variation in bone core length across specimens and the number of samples analyzed.

**TABLE 2:** Comparison of trabecular bone parameters of AIS and healthy (control) adolescents—bone volume fraction (BV/TV) and trabecular thickness (Tb.Th)—across studies.

Study	Country	Population	Age (years)	Method	Anatomical site	Group	BV/TV	Tb.Th (mm)
Current study	Australia	White	14.8 ± 1.5	μCT	Vertebral body	AIS	0.146 ± 0.029	0.146 ± 0.011
Wang et al. [16]	China	Chinese	14.8 ± 1.89	μCT	Iliac crest	AIS	0.180 ± 0.033	0.120 ± 0.002
Wang et al. [16]	China	Chinese	14.5 ± 4.13	μCT	Iliac crest	Control	0.190 ± 0.035	0.114 ± 0.001
Zhu et al. [23]	China	Not reported	14.9	μCT	Iliac crest	AIS	0.199 ± 0.034	0.156 ± 0.054
Zhu et al. [23]	China	Not reported	14.6	μCT	Iliac crest	Control	0.133 ± 0.030	0.079 ± 0.017
Yang et al. [22]	China	Chinese	12.1 ± 0.92	HR‐pQCT II	Distal radius	AIS	0.190 ± 0.032	0.214 ± 0.009
Wang et al. [20]	China	Chinese	12.7 ± 0.8	HR‐pQCT I	Distal radius	AIS	0.127 ± 0.022	0.073 ± 0.009
Wang et al. [20]	China	Chinese	12.7 ± 0.8	HR‐pQCT I	Distal radius	Control	0.128 ± 0.021	0.072 ± 0.008
Yu et al. [19]	China	Chinese	12.9 ± 0.6	HR‐pQCT I	Distal radius	AIS	0.124 ± 0.023	0.072 ± 0.009
Yu et al. [19]	China	Chinese	12.9 ± 0.6	HR‐pQCT I	Distal radius	Control	0.127 ± 0.021	0.072 ± 0.008
Yu et al. [18]	China	Chinese	13.0 ± 0.6	HR‐pQCT I	Distal radius	AIS	0.130 ± 0.020	0.074 ± 0.010
Wang et al. [16]	China	Chinese	14.8 ± 1.89	Histomorphometry	Iliac crest	AIS	0.265 ± 0.006	—
Wang et al. [16]	China	Chinese	14.5 ± 4.13	Histomorphometry	Iliac crest	Control	0.280 ± 0.007	—
Tanabe et al. [24]	Japan	Japanese	14.4	Histomorphometry	T12 vertebral process	AIS	0.124	0.117
Cheng et al. [17]	China	Not reported	14	Histomorphometry	Iliac crest	AIS	0.219 ± 0.018	—
Glorieux et al. [55]	Canada	Not reported	14.0–16.9	Histomorphometry	Iliac crest	Control	0.257 ± 0.053	0.157 ± 0.022

Since trabecular microarchitecture independently determines estimated bone strength [56, 58], we analyzed plate versus rod‐like trabeculae using the ellipsoid factor (Tb.EF). As shown in Figure 3b, Tb.EF showed no significant correlations with curve angle or vertebral wedging. Tb.EF values ranged from −0.1277 to 0.1588, spanning both negative and positive values, indicating that the trabecular microarchitecture did not show a consistent predominance of either plate‐like or rod‐like structures, as a Tb.EF value of zero corresponds to an intermediate or spherical geometry. Zhu et al. [23] reported that patients with congenital scoliosis had more plate‐like trabeculae than patients with AIS, although whether this characteristic is associated with the development or progression of AIS remains unclear. Notably, a more plate‐like trabecular architecture has been associated with greater estimated bone strength [58, 59], independent of BV/TV [60, 61]. Additionally, Tb.EF remained relatively constant with Risser grade, contrasting with previous studies showing trabeculae become more plate‐like with age [57, 62, 63, 64].

The Tb.Th values observed in our study ranged from 0.13 to 0.17 mm, which align closely with previous reports for AIS trabecular bone from the iliac crest (0.15 ± 0.05 mm [23]; 0.12 ± 0.0016 mm [16]). Consistent with the findings of Zhu et al. [23] and Wang et al. [16], our results also indicate slightly greater Tb.Th values than those reported for healthy controls. However, our values were notably higher than those reported for the distal radius (0.072–0.074 mm [18, 19, 20]), as shown in Table 2. The consistency between our results in the Australian/White population and studies of Chinese populations at the iliac crest suggests that the observed discrepancy with distal radius measurements may stem from two factors. First, methodological differences likely contribute: Yu et al. [18, 19] and Wang et al. [20] employed HR‐pQCT for evaluation, whereas Zhu et al. [23], Wang et al. [16], and our study utilized micro‐CT on bone biopsies. Second, anatomical differences between skeletal sites may contribute to these discrepancies, as trabecular microstructure characteristics can vary significantly between the iliac crest, vertebral bodies, and distal radius. Our analysis revealed no clear Risser grade‐related dependence, which contrasts with the findings of Mitchell et al. [57]. For context, Tb.Th in healthy adolescents (ages 8–18) ranges from 0.20 to 0.24 mm in the tibia [57]. In comparison, most AIS subjects in our study exhibited Tb.Th values below 0.15 mm, indicating reduced Tb.Th in AIS relative to the radii and tibiae of healthy controls [57]. It should be noted that while trabecular structure and thickness can vary with body weight [57, 65], this factor was not considered in the present analysis given the similar weights across our study population (mean 50.9 ± 7.5 kg).

**FIGURE A2:**
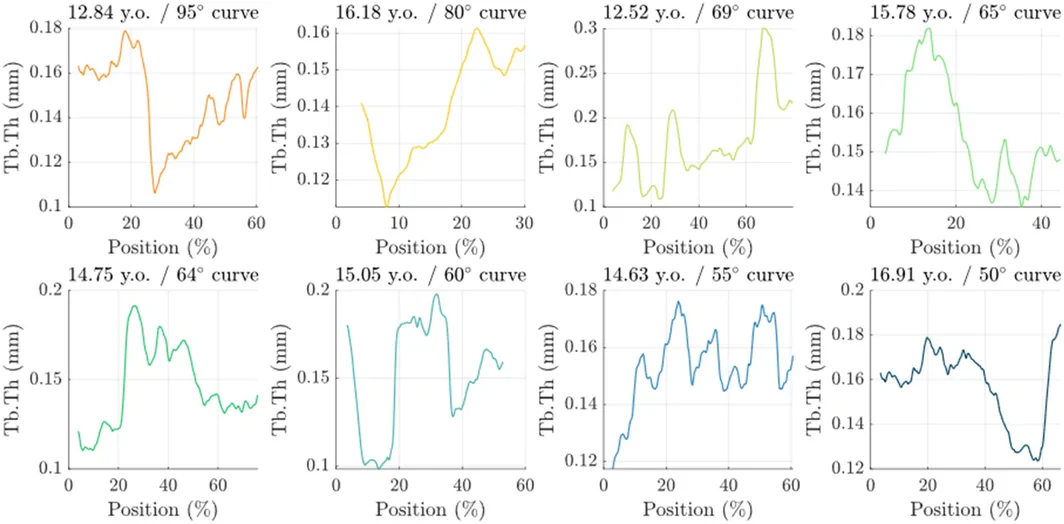
Trabecular thickness (Tb.Th) of bone specimens versus normalized vertebral body bone specimens' length for VOI radius size of *R* = 1.5 mm. Note that the graphs are ordered by value of curve angle, and the colors can be matched to the participants as described in Table 1.

**FIGURE A3:**
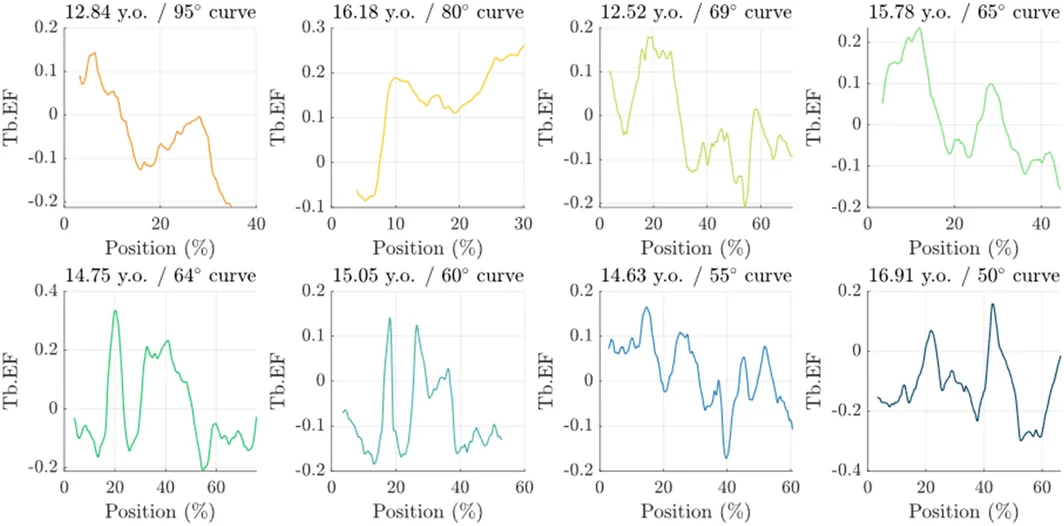
Ellipsoid factor (Tb.EF) of bone specimens versus normalized vertebral body bone specimens' length for VOI radius size of *R* = 1.5 mm. Note that the graphs are ordered by value of curve angle, and the colors can be matched to the participants as described in Table 1.

The significant positive correlation between Tb.Th and BV/TV and the negative association between Tb.EF and BV/TV align with previous reports, as thicker trabeculae are associated with increased bone volume. The interpretation of the ellipsoid factor is more complex, as this parameter captures the three‐dimensional geometry of the trabecular network. Nevertheless, plate‐like trabeculae (Tb.EF < 0) contribute substantially to bone architecture and have been associated with greater mechanical strength [59]. Regarding the spatial variation of Tb.Th and Tb.EF across the apical vertebral body, we found no consistent trend in any of the analyzed specimens for either parameter (see Figures A2 and A3). These findings suggest that region‐dependent bone adaptation (convex versus concave) is unlikely to be a dominant mechanism in AIS, although confirmation in a larger cohort with more standardized biopsy lengths is warranted.

The observed BMDD distribution curves of vertebral bone samples of our AIS subjects showed different patterns as the BMDD distribution curves of iliac crest bone biopsies for healthy young (aged 1.5–23 years) [37] and adult subjects (aged 25–90 years) [49] (Figure 4a). The mineralization peak (CaPeak) of the AIS vertebral body bone was closer to the iliac crest references of healthy adult bone (22.94 ± 0.39 wt% Ca) than of young bone (21.66 ± 0.52 wt% Ca), and the distribution's width (CaWidth) appears to be higher in the AIS vertebral body bone. (Figure 4b,c). Although CaPeak did not show a significant relationship with major curve angle, it exhibited a strong negative correlation with apical wedge angle (*p* < 0.01), suggesting two potentially important findings. First, the association between greater vertebral wedging and lower bone mineralization is consistent with the findings of Li et al. [66], who reported that lower bone mineral density was associated with an increased likelihood of curve progression. Second, the lack of a significant association between CaPeak and major curve angle, together with its strong association with apical wedge angle, suggests that major curve angle may capture aspects of spinal deformity beyond vertebral wedging. This interpretation is consistent with the findings of Modi et al. [38], who reported that major curve angle, particularly in the lumbar spine, may be influenced more strongly by disc wedging than by vertebral wedging. Moreover, CaPeak shows an increasing dependence on Risser grade and age (Spearman's *ρ* = 0.77, *p* = 0.02), which agrees with the trend observe on the young versus adult curves [37, 49] (seen in Figure 6a), although disagrees with other studies stating that the BMDD parameters of iliac trabecular bone are constant from 1.5 to 23 years of age [37, 67]. As discussed by Fratzl et al. [37], this lack of variation might be due to the positioning of their iliac biopsies around the growth plate, where we observe high bone turnover with a small positive balance towards new bone acquisition [68], resulting in young/newer bone. This does not hold for our AIS specimens, since they were extracted from the middle of the vertebra, further from the growth plates [69], and in a vertebral region where little to no growth is observed in this age range [70].

The CaWidth, on the other hand, appears to remain largely constant with both curve and apical wedge angles and Risser stage. Notably, these values remain elevated compared to those observed in healthy young and adult populations, suggesting that increased spinal deformity may be associated with greater heterogeneity in mineralization. This association is particularly significant given that age appears to have minimal influence on this parameter (Spearman's *ρ* = −0.35, *p* = 0.38), indicating that the observed mineralization heterogeneity is likely related to the pathological process rather than normal developmental changes. Other bone diseases associated with increased CaWidth include osteomalacia and osteoporosis [50]. Compared with these conditions, the elevated CaWidth in AIS vertebral body bone may be explained by the prevalence of osteoporosis and osteopenia in patients with scoliosis being greater than in the general population [17, 25, 71, 72]. Note that BMDD variations are usually explained by two main factors: turnover or mineralization kinetics [73]. In this case, the variation in BMDD parameters would be mainly attributed to abnormalities in bone turnover. This finding is consistent with the results of Zhang et al. [21], who observed elevated levels of systemic bone turnover markers and increased local bone remodeling in the distal radius of AIS patients, suggesting an association between disrupted bone turnover and the risk of AIS progression. However, to better correlate these results in terms of bone metabolism, histomorphometry analysis should be performed in the samples to analyze if these parameters are indeed associated with excessive osteoid deposition or rather high bone formation.

The biomechanical properties of the bone matrix are commonly assessed using nanoindentation. At the nanoindentation scale, measurements probe relatively large regions of the bone matrix that may contain lacunae as well as heterogeneity in BMDD, and therefore are less sensitive than qBEI analysis, which does not average the mineral distribution over larger areas. Our reduced modulus measurements averaged approximately 13 GPa, which is within the range reported for healthy bone. For comparison, average trabecular reduced moduli measured in adults' vertebra and skull using nanoindentation were 13.4 ± 2.0 GPa [54] and 13.85 ± 2.0 GPa [53], respectively. Nanoindentation measurements in 8‐year‐old subjects reported a reduced modulus of 8.77 GPa [53]. The hardness measurements were also consistent with published values. In the present study, hardness ranged from 0.30 to 0.52 GPa, while mean hardness values reported in adults and children were 0.46 ± 0.08 GPa [54] and 0.30 ± 0.01 GPa [53], respectively. Note that although Wang et al. [53] investigated skull bone rather than vertebral bone, their measurements in adults (mean age: 51 years; hardness: 450.4 ± 74 MPa; reduced modulus: 13.85 ± 2.0 GPa) were comparable to those reported by Rho et al. [54] for adult vertebral trabecular bone. Given the limited availability of pediatric vertebral nanoindentation data, we therefore consider the measurements from the 8‐year‐old subjects to provide a relevant reference for comparison.

The observed decrease in reduced modulus (and similar trends with hardness) with increasing apical vertebral wedging may be consistent with previous reports of altered bone quality in AIS. For example, Yang et al. [22] reported decreased cortical bone density and mechanical strength alongside elevated bone turnover markers in girls with AIS at peak height velocity. Similarly, AIS has been associated with lower failure load and apparent modulus [74]. In the present study, CaPeak was positively correlated with hardness and showed a positive trend with reduced modulus, suggesting a potential association between tissue mineralization and mechanical properties. Together, these findings may suggest that mechanical properties are heterogeneous in AIS and that tissue‐level mechanical alterations may be associated with the severity of local vertebral deformity rather than uniformly affecting all individuals with AIS. The association between reduced modulus and vertebral wedging, together with the relationship between mineralization and tissue‐level mechanical properties, may further support the hypothesis that altered bone quality during growth could contribute to the susceptibility of the vertebrae to deformation. However, given the small sample size and cross‐sectional nature of the present study, these findings should be considered exploratory, and longitudinal studies are needed to determine whether reduced mechanical properties contribute to vertebral wedging or develop as a consequence of the deformity. Consistent with Rho et al. [54, 75], the extracted reduced modulus exhibited high variability, which may mask more subtle trends. The lack of a significant correlation between major curve angle and reduced modulus further suggests that major curve angle may capture aspects of spinal deformity beyond vertebral wedging. Moreover, no significant correlation was observed between the reduced modulus and hardness versus the Risser grade, which contrasts with reported age‐related increases in bone density during sexual development [76, 77] and the increase in cortical bone stiffness observed in growing teenagers [78].

Finally, when analyzing bone strength (hardness and reduced modulus) in bone cores retrieved from apex vertebral bodies of AIS patients, we found no dependence of mechanical behavior on spatial position between convex and concave sides (see Appendix A). Note that an analysis technique such as that proposed by Pastrama et al. [79] could have delineated differences in bone matrix stiffness—as they observed in adult femurs from endosteum to periosteum—but this analysis would require a larger number of indentations to be performed.

This study has several limitations. The primary limitation of this study is the small sample size and its impact on the statistical analysis, including potential differences related to curve patterns, the use of two different surgical approaches, and variations in curve severity and patient age. However, given the challenges of collecting vertebral body bone cores during scoliosis surgery, we consider this dataset unique and valuable as a case study. Spatial variation analysis proved limited, partly due to sample variability. Note that the extraction procedure was standardized and well‐practiced, although samples fractured during extraction from the needle—which resulted in samples generally shorter than the full cortex‐to‐cortex width of the vertebral body—extreme care was taken to ensure that the bone cores were not compressed during the coring procedure, achieving the best possible outcomes despite challenges including reduced bone quality in adolescent patients, difficult surgical access via small lateral incisions (thoracolumbar scoliosis) or 3 cm thoracoscopic portals (thoracic scoliosis), and limitations of the bone biopsy tool (the best and longest available). Therefore, sample variability can be attributed to patient variability, small sample size, edge artifacts from unavoidable outer core damage, and inconsistent core extraction given the small diameter‐to‐length ratio. Micro‐CT analysis of BV/TV assumes extraction does not alter trabecular pore structure. While the T‐LOK manufacturer asserts pore space remains unaffected, some influence from needle insertion cannot be ruled out. However, Tb.Th measurements are unlikely affected by this artifact. Additionally, we lacked vertebral body bone from matched healthy adolescent controls for the same analysis and could only compare with controls from other studies and/or body locations. Correlative data from peripheral skeletal assessment, for example, DXA or HR‐pQCT, were also not included, since these imaging modalities are only performed clinically in cases of osteopenia, and are not a routine scan for scoliosis patients. Moreover, the apical wedge angle was measured from two‐dimensional radiographs. As scoliosis is a three‐dimensional spinal deformity, the measured wedge angle may underestimate the true extent of vertebral deformity; however, it still captures vertebral deformation in the imaged plane. Furthermore, the qBEI analysis is limited by lacking reference BMDD data for vertebral bone from healthy adolescents or adults. We relied on iliac crest biopsy reference data, which may introduce inconsistency due to site‐specific bone turnover differences affecting BMDD. Finally, the homogeneity of patient demographics presents another limitation that future work should address.

## 5 Conclusions

This study presents the first comprehensive analysis of trabecular bone quality and mechanical properties in vertebral body bone samples from AIS patients. To our knowledge, this is the first investigation assessing vertebral body bone quality in this population. Our main finding was that the mineralization peak (CaPeak) and width (CaWidth) of AIS apical vertebral bone exceeded values in healthy young populations, suggesting increased spinal deformity may be associated with greater mineral heterogeneity. These results support the hypothesis that AIS patients exhibit altered bone quality and biomechanical properties. The observed trabecular microarchitecture and mineralization pattern changes in vertebral bodies may contribute to AIS pathogenesis and spinal deformity progression. Future work should extend the present study through a multimodal experimental approach by complementing the existing mineralization and microstructural analyses with histomorphometric assessment of cellular activity and blood biomarker analyses to better understand the mechanisms underlying the observed bone alterations.

## Author Contributions

**Natalia M. Castoldi:** conceptualization, methodology, data curation, software, validation, formal analysis, investigation, visualization, writing – review and editing, writing – original draft, project administration. **James Roberts:** investigation, data curation, visualization. **Davide Fontanarosa:** methodology, software, formal analysis, writing – review and editing, supervision. **Geoffrey N. Askin:** conceptualization, methodology, writing – review and editing, formal analysis. **Robert D. Labrom:** conceptualization, methodology, formal analysis, writing – review and editing. **Jurgis Ruza:** investigation, data curation, writing – review and editing. **Jiaqiu Wang:** methodology, validation, formal analysis, investigation, writing – review and editing. **Maree T. Izatt:** conceptualization, methodology, writing – review and editing, supervision, formal analysis. **Peter Pivonka:** conceptualization, methodology, formal analysis, resources, writing – review and editing, supervision, project administration, funding acquisition. **Edmund Pickering:** conceptualization, methodology, software, validation, formal analysis, investigation, writing – original draft, writing – review and editing, data curation, visualization, project administration. **Madge Martin:** conceptualization, funding acquisition, methodology, validation, formal analysis, writing – review and editing, supervision. **J. Paige Little:** conceptualization, methodology, formal analysis, supervision, writing – review and editing, funding acquisition. **Laure Stickel:** investigation, data curation, writing – review and editing.

## Funding

This work was supported by Australian Research Council (IC190100020, DP230101404). L'Oréal‐UNESCO For Women in Science Fellowship.

## Conflicts of Interest

The authors declare no conflicts of interest.

## Figures and Tables

**FIGURE A4:**
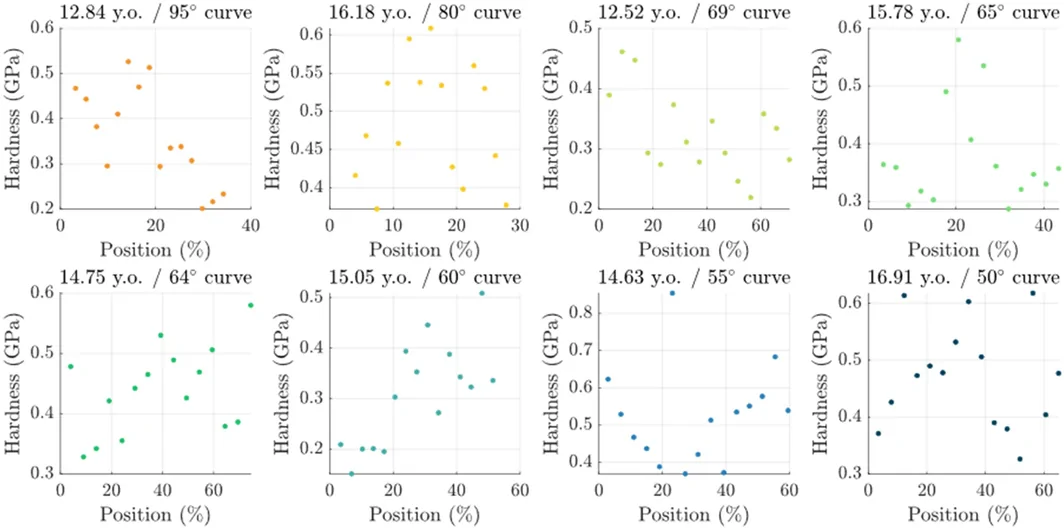
Hardness of bone specimens versus normalized vertebral body bone specimens' length. Note that the graphs are ordered by value of curve angle, and the colors can be matched to the participants as described in Table 1.

**FIGURE A5:**
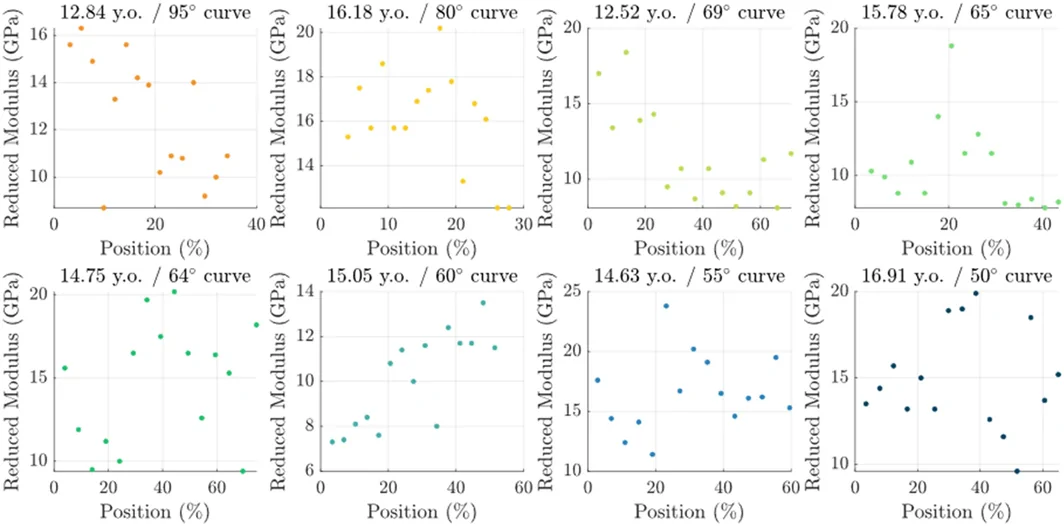
Reduced modulus of bone specimens versus normalized specimens' length. Note that the graphs are ordered by value of curve angle, and the colors can be matched to the patients as described in Table 1.

## Data Availability

The data that support the findings of this study are available on request from the corresponding author. The data are not publicly available due to privacy or ethical restrictions.

## Appendix A

### Analysis of Variables Along the Convex–Concave Axis

In this section, we present the raw results along the length of the AIS vertebral body bone specimens, moving from the convex side to the concave side (i.e., from lower to higher mechanical loading). We could not guarantee that all bone cores were bicortical; therefore, the extracted specimens were generally shorter than the full cortex‐to‐cortex width of the vertebral body. Care was taken to ensure that the bone cores were not compressed during the coring procedure. However, some specimens fractured during extraction or removal from the coring needle. In these cases, only the intact, undamaged portions of the specimens were used for subsequent analyses. Since the exact fracture location along the core could not be determined, all specimens were aligned using the convex side as a reference (0%), and their position was expressed as a percentage of the estimated vertebral body width, which was estimated from post‐surgical X‐rays using the surgical screws as reference dimensions.

Figure A1 shows the bone volume fraction (BV/TV) obtained from micro‐CT scans for two different volumes of interest (VOI) radii: *R* = 1 mm and *R* = 1.5 mm. Based on our analysis, a VOI radius of approximately 1 mm provides a robust and reliable measure of BV/TV. In contrast, values obtained from VOIs with *R <* 1 mm are not representative. This finding is particularly relevant for bone biopsy studies that use biopsy gauges smaller than 2 mm. No consistent trend in BV/TV variation was observed along the length of the specimens.

The evolution of Tb.Th along the specimen length seems to increase in some samples from the convex side (lower mechanical loading) to the concave side (higher mechanical loading), as shown in Figure A2. However, this trend is not consistently observed and should be interpreted with caution due to the variation in bone sample lengths.

We observe a decrease in the ellipsoid factor in the majority of samples (see Figure A3). This suggests an evolution of bone microarchitecture from rod‐like structures in the convex region (lower mechanical loading) to platelike structures in the concave region (higher mechanical loading). However, this trend is not consistently observed and should be interpreted with caution due to variations in bone sample length.

The mechanical analysis through nanoindentation is shown in Figures A4 and A5. No clear trends in hardness or reduced modulus are observed along the length of the specimen. It is important to note that the positions of the measurements on the graph are not exact, as we only categorize the measurements into three regions: Concave, middle, and convex. In the graph, the measurements are equally distributed along the normalized length.
